# Supplementary figures and images for: Octadecaneuropeptide, ODN, Promotes Cell Survival against 6-OHDA-Induced Oxidative Stress and Apoptosis by Modulating the Expression of miR-34b, miR-29a, and miR-21in Cultured Astrocytes
Source: Cells. 2024 Jul 12;13(14):1188. doi: 10.3390/cells13141188 (PMC11487398; doi:10.3390/cells13141188)

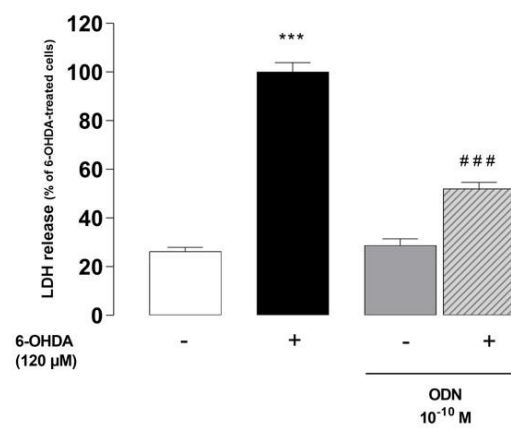

Supplementary Figure 1  
Bourzam et al. 2024

Supplement: Supplementary file 1 [file cells-13-01188-s001.zip › cells-3075061-supplementary.pdf]
